# Orange photons (623 nm) resulted in similar or greater lettuce growth than red photons (660 nm): comparative effects on morphology, photon capture, and photosynthesis

**DOI:** 10.3389/fpls.2025.1653524

**Published:** 2025-07-30

**Authors:** Seonghwan Kang, Shuyang Zhen

**Affiliations:** Department of Horticultural Sciences, Texas A&M University, College Station, TX, United States

**Keywords:** indoor farming, light spectral quality, yield, photosynthetic efficiency, cryptochromes, photomorphogenesis

## Abstract

Photosynthetic efficiency is wavelength-dependent. Previous studies found that photons between ~600–625 nm (herein referred to as ‘orange photons’) resulted in the highest quantum yield (i.e., moles of CO_2_ fixed per mole of absorbed photons), followed by ~650–675 nm red photons. However, these findings were based on short-term, single-leaf measurements, and limited information is available on the long-term effects of orange photons on plant growth and photosynthesis. Orange photons may differentially influence photoreceptors such as cryptochromes and phytochromes compared to red photons, leading to changes in plant morphology and phytochemical accumulation. Therefore, our objective was to quantify the effects of orange versus red photons on plant growth, morphology, and photosynthetic responses. Two lettuce cultivars, green butterhead ‘Rex’ and red oakleaf ‘Rouxai’, were grown in a growth chamber under four light spectral treatments using blue (B; 444 nm), green (G; 536 nm), orange (O; 623 nm), red (R; 660 nm), and far-red (FR; 730 nm) light emitting diodes (LEDs): 1) B_50_G_25_O_175_, 2) B_50_G_25_R_175_, 3) B_50_G_25_O_137.5_FR_37.5_ (O+FR), and 4) B_50_G_25_R_137.5_FR_37.5_ (R+FR). Subscripts indicate photon flux density in µmol m^-2^ s^-1^; all treatments had the same total photon flux density of 250 µmol m^-2^ s^-1^. Orange photons generally resulted in similar or greater plant growth than red photons. Specifically, in the absence of FR, replacing red with orange photons increased total leaf area and shoot dry weight in ‘Rex’ by 12-15%, likely resulting from cryptochrome deactivation. In contrast, orange photons reduced anthocyanin accumulation in red lettuce ‘Rouxai’ without affecting yield. The inclusion of FR photons significantly increased leaf area and shoot biomass in both cultivars, with similar growth observed under the O+FR and R+FR treatments. While leaf photosynthesis rate of ‘Rex’ was lower under orange measurement light on an incident photon basis, quantum yield was generally higher under orange than red light. Given that current orange LEDs are less energy-efficient than red LEDs, it is important to consider both the plant growth benefits and energy costs when using orange photons in controlled environments.

## 1 Introduction

Crop lighting with narrow-spectrum light-emitting diodes (LEDs) in controlled environment agriculture (CEA) enables precise spectral control to optimize plant growth, morphology, physiology, and productivity. Currently, the most commonly used LED fixtures for horticultural lighting have emission peaks primarily in the blue (around 450 nm) and red (around 660 nm) regions of the photosynthetically active radiation (PAR; 400–700 nm) range. These fixtures are highly energy-efficient, and their spectral outputs closely match the absorption peaks of chlorophylls, thereby maximizing photosynthetic photon absorption (Okamoto et al., 1996; Massa et al., 2008; Kusuma et al., 2021).

Photosynthetic efficiency is wavelength-dependent. Studies by McCree (1971) and Inada (1976) demonstrated that photons in the ~600–625 nm spectral region resulted in the highest quantum yield, i.e., moles of CO_2_ fixed per mole of absorbed photons, within the PAR region across various species and cultivars. These photons are referred to as orange photons herein, based on their perceived color, to distinguish them from the commonly used red photons provided by standard red LED fixtures (peak ~660 nm). Although the quantum yield of orange photons was approximately 10% higher than that of the most efficient red photons in the ~650–675 nm region (McCree, 1971), leaf photosynthetic rate may be lower under orange photons than under red photons on an incident photon basis due to less efficient leaf light absorption in the orange region. For instance, Massa et al. (2015) reported significantly higher leaf transmittance of orange photons (peak at 631 nm) compared to red photons (peak at 655 nm) in lettuce and cucumber. Nonetheless, the lower leaf absorptance in the orange region can result in deeper light penetration into the leaf tissue and plant canopy, thereby improving canopy light distribution and potentially enhancing biomass production at the whole-plant level (Massa et al., 2015; Dieleman et al., 2019). Dieleman et al. (2019) observed greater shoot dry weight in greenhouse-grown tomatoes supplemented with orange photons (627 nm) compared to red photons (660 nm), likely due to better light distribution inside the canopy. Deeper light penetration by orange photons may help sustain photosynthesis in lower canopy layers, contributing to overall biomass gains.

Despite the potential of orange photons to elicit high photosynthetic activity, most previous studies have only examined short-term leaf-level photosynthetic responses or have been conducted over short cultivation periods, primarily focusing on morphological traits during the seedling stage (McCree, 1971; Inada, 1976; Wollaeger and Runkle, 2013; Massa et al., 2015; Dieleman et al., 2019). The long-term effects of orange photons on plant growth, morphology, physiology, and phytochemical composition remain unclear.

Orange photons may influence plant morphology and phytochemical accumulation through the de-activation of cryptochromes (Bouly et al., 2007; Battle et al., 2020). Cryptochromes are plant photoreceptors that primarily sense ultraviolet-A (UV-A), blue, and green light (Más et al., 2000; Banerjee and Batschauer, 2005; Bouly et al., 2007). Active cryptochromes mediate various morphological and phytochemical responses, including the inhibition of stem elongation and leaf expansion, as well as the enhancement of phytochemical accumulation across a wide range of species/cultivars (Meng et al., 2019; Kusuma et al., 2021; Kang et al., 2022; Zhu et al., 2024). Conversely, de-activation of cryptochromes promotes stem elongation and leaf expansion, often leading to increased photon capture and biomass accumulation (Meng et al., 2019; Kusuma et al., 2021). However, the concentrations of beneficial phytochemicals, such as anthocyanins and phenolic compounds, are generally reduced under conditions in which cryptochromes are de-activated (Meng et al., 2019; Kusuma et al., 2021; Kang et al., 2022; Zhu et al., 2024). Light in the ~530–630 nm spectral region, which includes green, yellow, and orange photons, has been shown to de-activate cryptochromes (Bouly et al., 2007; Battle et al., 2020). Therefore, orange photons likely induce changes in plant growth, morphology, and phytochemical content during long-term crop cultivation.

In addition to their effects on cryptochromes, orange photons may affect the activation state of phytochrome photoreceptors differently than red photons. Phytochromes primarily sense red (absorption peak ~670 nm) and far-red (~730 nm) light (Casal, 2012; Gommers et al., 2013; Zhen et al., 2022). Like cryptochromes, phytochromes play a critical role in regulating plant growth, morphology, and the accumulation of phytochemicals. Red light activates phytochromes, whereas far-red light serves as a shade signal and deactivates phytochromes, triggering shade avoidance or tolerance responses such as reduced leaf thickness, increased stem elongation, and changes in leaf expansion (Casal, 2012; Gommers et al., 2013). Previous studies have shown that green photons—which de-activate cryptochromes and induce shade-like responses—can enhance far-red-induced shade responses (Wang et al., 2015). Since orange photons also de-activate cryptochromes, orange photons may act synergistically with far-red photons to regulate plant growth and shade responses. The interactions between orange and far-red photons, in comparison to red photons, need to be examined when considering replacing or partially replacing red with orange photons for crop lighting.

Therefore, the objectives of this study were to: 1). quantify the long-term effects of orange photons on plant growth, photosynthesis, morphology, leaf optical properties, and pigment composition, and 2) examine how orange versus red photons interact with far-red photons to regulate plant growth, morphology, and pigmentation. Two economically important lettuce cultivars—green butterhead lettuce ‘Rex’ and red oakleaf lettuce ‘Rouxai’—were used to assess potential cultivar-specific responses. Findings from this study will support the optimization of light spectral quality for LED-based crop production in controlled environments.

## 2 Materials and methods

### 2.1 Plant materials

Two lettuce cultivars, green butterhead lettuce (*Lactuca sativa*) ‘Rex’ and red oakleaf lettuce ‘Rouxai’ were used in this study. Seeds were sown in 1.5 L plastic containers (10.8 cm × 10.8 cm × 12.5 cm; length × width × height) filled with a commercial peat-based substrate (LM-111; Lambert Peat Moss Co., Riviere-Quelle, Quebec, Canada). After sowing the seeds, the containers (96 total; 48 per cultivar) were moved into a walk-in growth chamber (model BDW40; CONVIRON, Winnipeg, MB, CANADA) with inner dimensions of 2.36 m (length) × 1.57 m (depth) × 2.41 m (height). Immediately after sowing, four light spectral treatments were initiated (described in detail below). The growth chamber was divided into four sections using two vertical growing racks, each with two layers. Each section (1.2 m × 0.6 m × 1.1 m; length × depth × height) was randomly assigned and subjected to one of four light spectral treatments. Reflective foils were used to cover the sides of each section and line the growing racks to minimize light contamination between sections. Two small air circulation fans (76.9 CFM; model CFM-9238B-140-473; Mouser Electronics, Mansfield, TX, USA) were installed on either side of each treatment section to increase air flow rate. Seedlings emerged three days after sowing (DAS). At six DAS, the seedlings of both cultivars were selected for uniformity and thinned to one plant per container. Eight experimental plants per cultivar were placed under each light treatment, with an additional four plants per cultivar placed along the edges as border plants to mitigate edge effects—resulting in a total of 24 plants in each treatment area (12 plants per cultivar).

### 2.2 Light treatments and growing conditions

**Figure 1:**
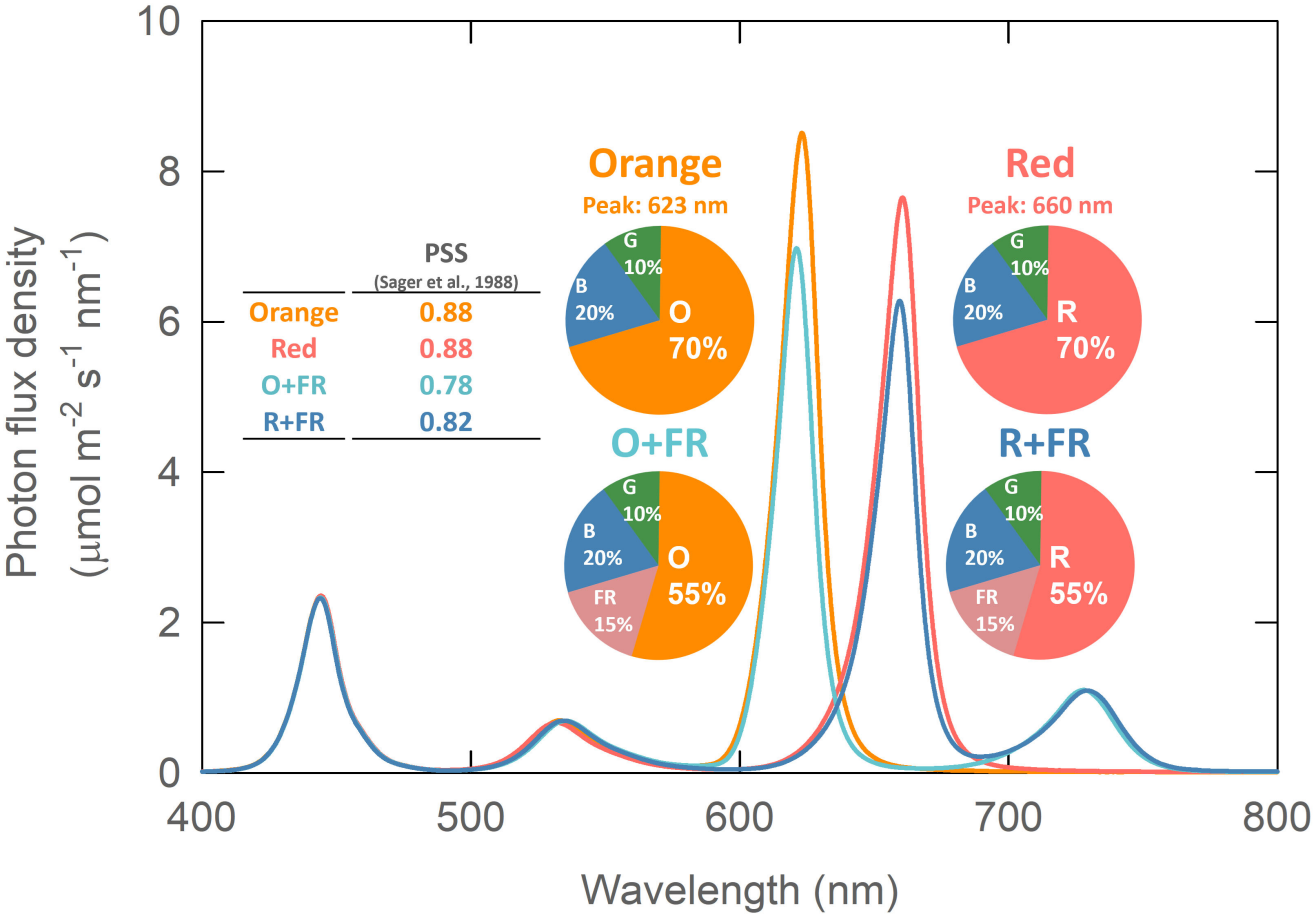
Spectral photon distribution of the four light treatments: Orange (B_50_G_25_O_175_), Red (B_50_G_25_R_175_), O+FR (B_50_G_25_O_137.5_FR_37.5_), and R+FR (B_50_G_25_R_137.5_FR_37.5_). B, G, O, R, and FR stand for blue, green, orange, red, and far-red light, respectively. The subscript numbers indicate the photon flux density of each waveband in µmol m^-2^ s^-1^. Phytochrome photostationary state (PSS) was calculated following the method described by Sager et al. (1988).

Four light spectral treatments were created using monochromatic LED bars—blue (B, peak at 444 nm), green (G, 536 nm), orange (O, 623 nm), red (R, 660 nm), and far-red (FR, 730 nm)—mounted about 60 cm above the plant canopy. The different color LED bars were installed in an alternating pattern (e.g., R-B-G-R-B-G-R) to improve spectral uniformity across the treatment area. The B, G, R, and FR LEDs were manufactured by Fluence Bioengineering (model Ray 22; Austin, TX, USA) and the orange LEDs were custom made by TCP Lighting (Cleveland, OH, USA). The four spectral treatments were as follows: 1) B_50_G_25_O_175_ (Orange), 2) B_50_G_25_R_175_ (Red), 3) B_50_G_25_O_137.5_FR_37.5_ (O+FR), and 4) B_50_G_25_R_137.5_FR_37.5_ (R+FR). Subscript indicates the flux density of photons emitted by each type of LED in µmol m^-2^ s^-1^. Note that all four treatments had the same total photon flux density (TPFD) of 250 µmol m^-2^ s^-1^, integrated from 400 to 800 nm. The fraction of photons emitted by the blue LEDs was 20% of the TPFD in all four treatments, while green LEDs contributed 10% (**Supplementary Table 1**). In two of the treatments, orange or red photons were partially substituted with FR photons. The light intensity and spectral distribution were measured at 16 locations within each treatment area using a spectroradiometer (PS300; Apogee Instruments, Logan, UT, USA) positioned 60 cm below the LEDs (**Figure 1**; **Supplementary Table 1**). The photoperiod was 16/8 hr (day/night). To minimize potential variations in light intensity and spectral ratios within each treatment, experimental plants were rotated daily. A constant distance of 60 cm between the light sources and the plant canopy was maintained by placing the plants on stacked plastic containers, which were gradually removed as the plants grew. The phytochrome photostationary state (PSS) was calculated following the method described by Sager et al. (1988) (**Figure 1**). The PSS values were identical (0.88) for the orange and red treatments (**Figure 1**, inset table). However, in the FR-substituted treatments, the O+FR treatment had the lowest PSS value (0.78), followed by the R+FR treatment (0.82).

Plants were fertigated daily with a water-soluble fertilizer solution containing 100 mg L^-1^ N and other essential nutrients (21N - 2.2P - 16.6K; 21-5–20 Peter’s Professional General Purpose; ICL Specialty Fertilizer, Summerville, SC, USA). The electrical conductivity of the nutrient solution was 1.2 dS m^-1^, and the pH was adjusted to 5.8. The environmental conditions inside the chamber were recorded using a temperature and humidity sensor (EE08-SS; Apogee Instruments, Logan, UT, USA) connected to a data logger (CR1000X; Campbell Scientific, Logan, UT, USA). During the experiment period, the average chamber air temperature was 23.0 ± 0.2°C (day) and 20.2 ± 0.2°C (night). The relative humidity and vapor pressure deficit (VPD) were 70.3 ± 2.5% and 0.80 ± 0.15 kPa, respectively. Additionally, type-J thermocouples were installed to monitor air temperature in each treatment. The average day (night) air temperatures in the two replications were as follows: 24.1 ± 0.4°C (20.0 ± 0.3°C) for the orange treatment, 24.1 ± 0.5°C (20.0 ± 0.3°C) for the red treatment, 24.1 ± 0.5°C (19.9 ± 0.2°C) for the O+FR treatment, and 23.9 ± 0.5°C (20.0 ± 0.3°C) for the R+FR treatment.

### 2.3 Growth parameters

Plants were harvested at two growth stages: young plant stage at 26 DAS for both cultivars and mature plant stage at 33 DAS for green lettuce and 36 DAS for red lettuce. Four plants per cultivar were harvested at each growth stage. For both cultivars, the following growth parameters were measured at both harvests: shoot fresh weight, shoot dry weight (after fully dried for 7 days at 80 °C in a drying oven), total leaf area (measured with a leaf area meter; model LI-3000C; LI-COR, Lincoln, NE, USA), and leaf mass per area [LMA; estimated by dividing shoot dry weight by total leaf area (g m^-2^)]. Leaf chlorophyll content (µmol m^-2^) of recently mature leaves in green lettuce was measured in the morning on each harvest day using a chlorophyll meter (MC-100; Apogee Instruments). Three leaves were selected per plant, and one measurement was taken from the light-exposed area of each leaf. The measurements were averaged for each plant before analysis.

### 2.4 Projected leaf area

Top-down photos of individual plants were taken every two days starting from 7 DAS. The photos were captured inside a customized photo booth illuminated by three 60-cm white LED bars, using a digital camera placed 140 cm above the plant canopy. Each plant was positioned at the center of a whiteboard (60 cm × 80 cm). The photos were analyzed using ImageJ image processing software to determine the projected leaf area (PLA).

### 2.5 Anthocyanin content index

Top-down photos of red lettuce plants, taken at each harvest using the same method described above, were analyzed to quantify the normalized difference anthocyanin index (NDAI) (**Supplementary Figure 1**). NDAI serves as an estimate of the average anthocyanin content within the projected plant canopy area and was calculated using a Python program developed by Kim and van Iersel (2023). The NDAI values are determined based on the optical properties of anthocyanins, which show high absorptance in the green region and low absorptance in the red region of the light spectrum. The Python program separates the plant objects from the background and extracts the pixel intensity of the green and red color channels from the RGB images to calculate the NDAI as (I_red_ – I_green_)/(I_red_ + I_green_), where I is the pixel intensity (Kim and van Iersel, 2023).

### 2.6 Leaf light absorptance

Leaf light absorptance in both lettuce cultivars was determined at the end of the experiment (mature plant stage) using a method similar to that described by Zhen et al. (2019). Measurements were made using the uppermost fully expanded leaves from three plants per spectral treatment per cultivar. UV-A, white, and far-red LEDs were installed in a dark room to create a broad-spectrum light source. The spectral distribution of the combined LED light was measured with a spectroradiometer (PS300, Apogee Instruments) positioned directly beneath the LEDs, serving as the reference for leaf transmittance measurements. Then, each selected leaf was placed above the spectroradiometer with its adaxial surface facing the LEDs; the transmitted light intensity and spectral distribution were recorded at a spectral resolution of 1 nm. Leaf reflectance was measured on the adaxial side of the leaves using a reflectance standard (AS-004, Apogee Instruments) and a reflectance probe (AS-003, Apogee Instruments) connected to the spectroradiometer (PS300, Apogee Instruments). Leaf absorptance was then calculated as:


Leaf absorptance=1−reflectance−transmittance

### 2.7 Leaf photosynthesis measurements

Leaf photosynthesis measurements were made on the green lettuce grown under all four spectral treatments to assess the photosynthetic efficiency of red *versus* orange photons. Red lettuce was not measured, as the photosynthetic responses would be affected by the non-uniform distribution of anthocyanins in the leaves, making it challenging to accurately compare the photosynthetic efficiency of red and orange photons. Six days prior to the final harvest, gas exchange measurements were conducted on four plants per treatment using a portable gas exchange system (LI-6800; LI-COR, Lincoln, NE) with a clear-top leaf cuvette chamber (6800-12A; LI-COR). Measurements were taken on recently matured leaves under four light spectral conditions: 1) monochromatic orange light (O_250_), 2) monochromatic red light (R_250_), 3) a combination of blue, green, and orange light (B_50_G_25_O_175_), and 4) a combination of blue, green, and red light (B_50_G_25_R_175_). The subscripts indicate the flux density of photons emitted by each type of LED in µmol m^-2^ s^-1^. All measurement spectral conditions had the same light intensity as the experimental treatments during plant growth, which was 250 µmol m^-2^ s^-1^. Environmental conditions inside the leaf cuvette chamber were maintained at an air temperature of 25 °C, a CO_2_ concentration of 400 μmol m^-2^ s^-1^, a VPD of 1.0 kPa, and an airflow rate of 600 μmol s^-1^. Measurements were initially taken under monochromatic orange or red light in random order, followed by the two mixed light conditions, also applied in random order. Plants were given 45 to 75 minutes under each spectral condition for stomatal conductance (g_s_) to reach a steady state. Leaf net photosynthetic rate (P_net_) and transpiration rate (E) were recorded. Lastly, dark respiration rate was determined after 30 minutes of darkness. Water use efficiency (WUE; moles of CO_2_ assimilated per mole of water transpired) was calculated as the ratio of P_net_/E. Following the measurements, plants were returned to their respective spectral treatments and remained under treatment conditions until final harvest.

### 2.8 Quantum yield estimation

Quantum yield for CO_2_ assimilation was estimated using the following equation: quantum yield = gross photosynthesis rate/absorbed PPFD. The gross photosynthesis rate under monochromatic orange or red light at a PPFD of 250 µmol m^-2^ s^-1^ was calculated as the sum of net photosynthesis rate and the absolute value of the dark respiration rate. The absorbed PPFD was calculated by multiplying the leaf absorptance spectrum (measured as described in Section 2.6) by the incident light spectral distribution of orange or red light, and then integrating the product over the spectral output range (570–700 nm for orange light and 600–720 nm for red light).

### 2.9 Experimental design and statistical analysis

This experiment was performed twice. Treatments were arranged in a randomized complete block design with four light spectral treatments, and each replicate study was treated as a block. In the second replicate study, the locations of the four spectral treatments were re-randomized. Bartlett’s test and the Shapiro-Wilk test were used to test homogeneity and normality of the residuals, respectively, for all data. When the assumptions were not met, the data were square root-transformed, and the tests on residuals were repeated. Projected leaf area data were fitted using a three-parameter sigmoidal regression function in SigmaPlot (Systat Software, San Jose, CA, USA). Data from each cultivar were analyzed separately using analysis of variance (ANOVA) in Statistical Analysis System (SAS Institute, Cary, NC). Mean separation among the treatments was performed using Duncan’s multiple range test, with statistical significance set at *P* < 0.05.

## 3 Results

### 3.1 Biomass and plant morphology at two growth stages

**Figure 2:**
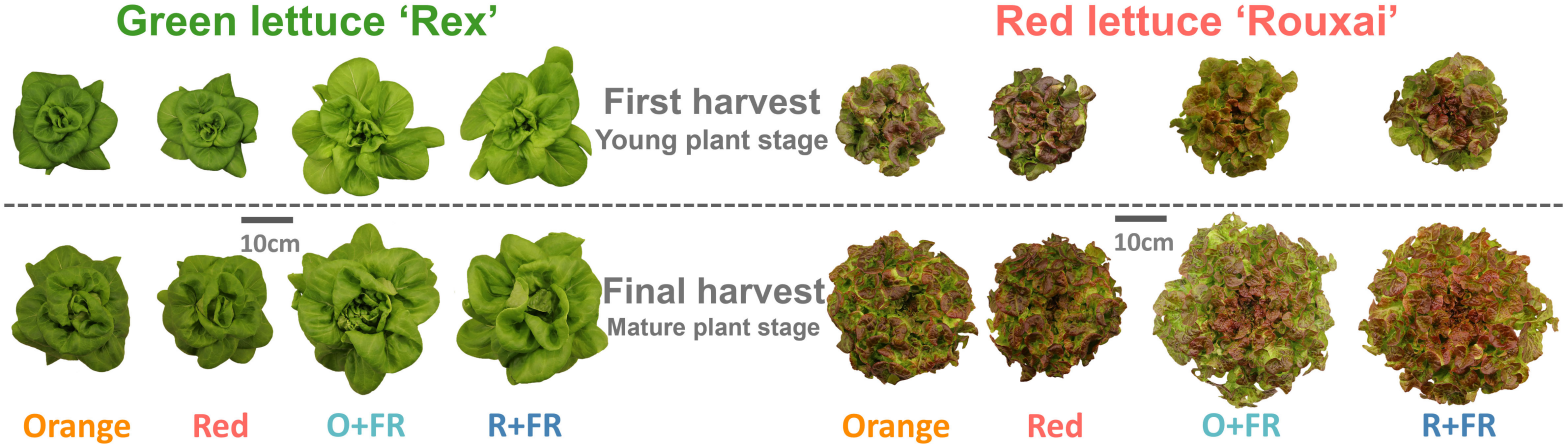
Representative plants of green lettuce ‘Rex’ and red lettuce ‘Rouxai’ grown under four light spectral treatments: Orange (B_50_G_25_O_175_), Red (B_50_G_25_R_175_), O+FR (B_50_G_25_O_137.5_FR_37.5_), and R+FR (B_50_G_25_R_137.5_FR_37.5_). B, G, O, R, and FR stand for blue, green, orange, red, and far-red light, respectively. The subscript numbers indicate the photon flux density of each waveband in µmol m^-2^ s^-1^. Plants were harvested at two growth stages: young plant stage (26 days after sowing) and mature plant stage (33 days after sowing for green lettuce and 36 days after sowing for red lettuce).

**Figure 3:**
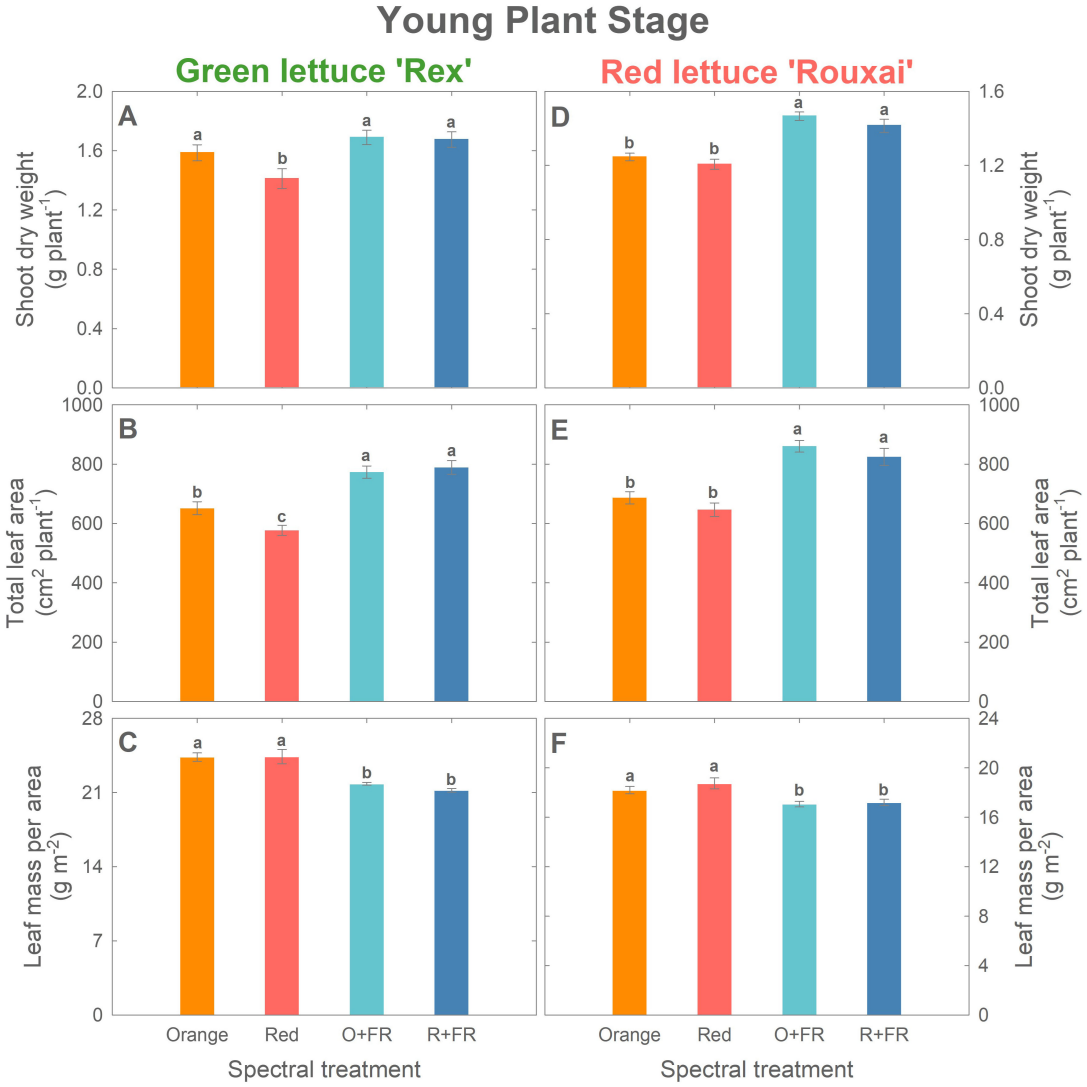
Growth parameters at the young plant stage (26 days after sowing): shoot dry weight **(A, D)**, total leaf area **(B, E)**, and leaf mass per area **(C, F)** of green lettuce ‘Rex’ **(A–C)** and red lettuce ‘Rouxai’ **(D–F)**. Plants were grown under four light spectral treatments: Orange (B_50_G_25_O_175_), Red (B_50_G_25_R_175_), O+FR (B_50_G_25_O_137.5_FR_37.5_), and R+FR (B_50_G_25_R_137.5_FR_37.5_). B, G, O, R, and FR stand for blue, green, orange, red, and far-red light, respectively. The subscript numbers indicate the photon flux density of each waveband in µmol m^-2^ s^-1^. Data represent mean ± SE (*n* = 8; 4 plants per treatment per harvest × 2 replications). Different letters denote significant treatment differences according to Duncan’s multiple range test at *P <* 0.05.

**Figure 4:**
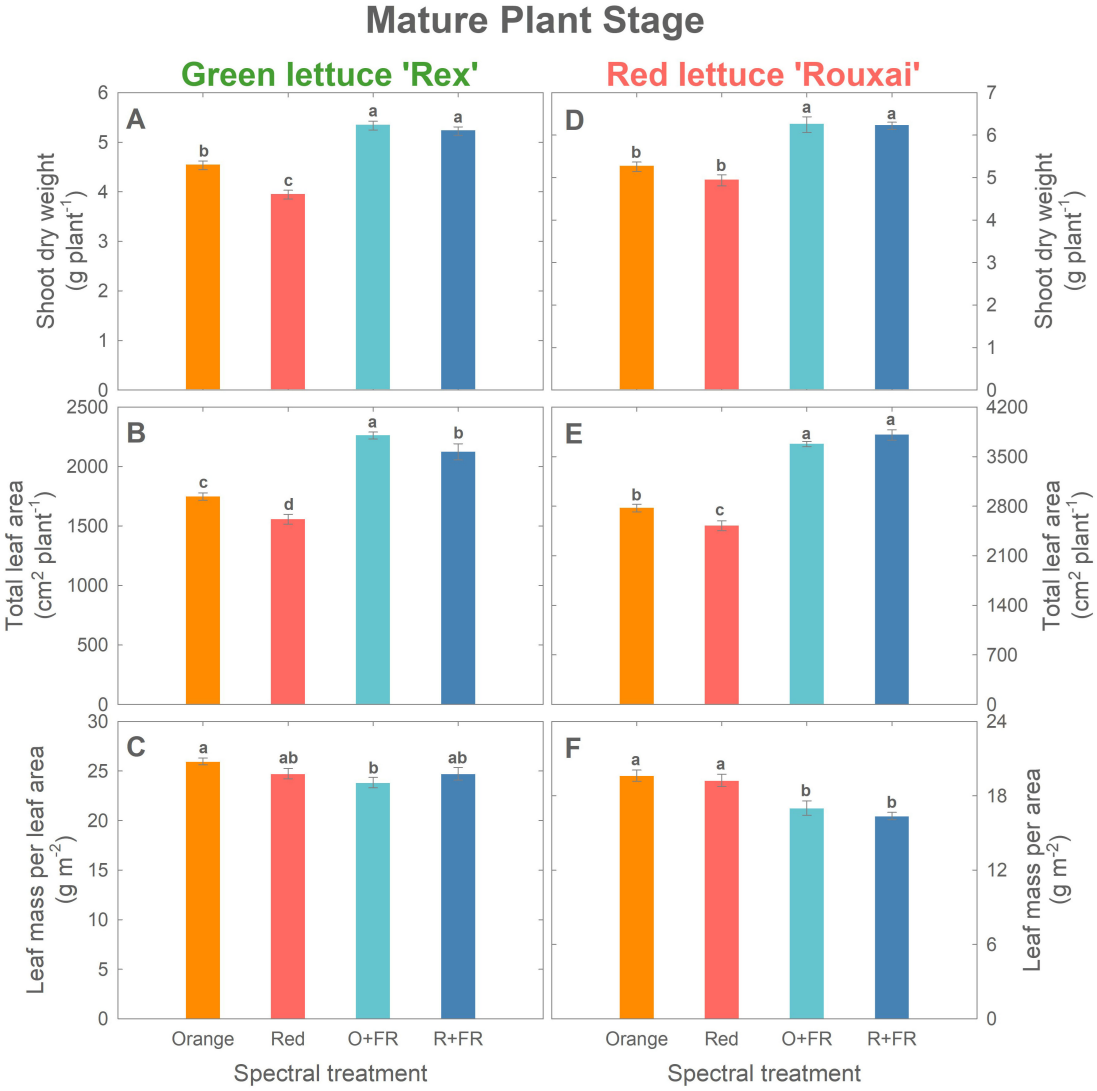
Growth parameters at the mature plant stage (33 days after sowing for green lettuce and 36 days after sowing for red lettuce): shoot dry weight **(A, D)**, total leaf area **(B, E)**, and leaf mass per area **(C, F)** of green lettuce ‘Rex’ **(A–C)** and red lettuce ‘Rouxai’ **(D-F)**. Plants were grown under four light spectral treatments: Orange (B_50_G_25_O_175_), Red (B_50_G_25_R_175_), O+FR (B_50_G_25_O_137.5_FR_37.5_), and R+FR (B_50_G_25_R_137.5_FR_37.5_). B, G, O, R, and FR stand for blue, green, orange, red, and far-red light, respectively. The subscript numbers indicate the photon flux density of each waveband in µmol m^-2^ s^-1^. Data represent mean ± SE (*n* = 8; 4 plants per treatment per harvest × 2 replications). Different letters denote significant treatment differences according to Duncan’s multiple range test at *P <* 0.05.

Plant biomass increased by more than twofold in green lettuce ‘Rex’ and over threefold in red lettuce ‘Rouxai’ between the young plant stage (26 DAS) and the mature plant stage (33 DAS for green lettuce and 36 DAS for red lettuce) (**Figures 2**–**4**; **Supplementary Figure 2**). Within each cultivar, the effects of spectral treatments on plant biomass accumulation and leaf expansion were generally consistent across both growth stages (**Figures 3**, **4**). Specifically, at the young plant stage, the orange spectral treatment resulted in significantly higher shoot dry weight (by 12%) and total leaf area (by 13%) in green lettuce ‘Rex’ compared to the red spectral treatment (**Figures 3A, B**). However, there was no significant difference in LMA between the orange and red spectral treatments (**Figure 3C**). Within the two FR-substituted treatments (O+FR and R+FR), no significant differences were observed in shoot fresh and dry weights, total leaf area, or LMA, even though the O+FR treatment had a lower PSS value (0.78) compared to the R+FR treatment (0.82) (**Figures 3A–C**; **Supplementary Figure 2A**). Both FR-substituted treatments resulted in significantly higher total leaf area but lower LMA (i.e., thinner leaves) compared to the red and orange spectral treatments (**Figures 3B, C**). Shoot dry weights in the FR-substituted treatments were higher than in the red treatment but similar to that in the orange treatment (**Figure 3A**).

In red lettuce harvested at the young plant stage, replacing red photons with orange photons had no significant effects on shoot dry weight, total leaf area, and LMA, regardless of FR substitution (**Figures 3D–F**). However, the two FR-substituted treatments yielded higher shoot dry weight and total leaf area, but lower LMA, compared to the treatments without FR photons.

At final harvest (mature stage; **Figure 4**), both cultivars exhibited growth patterns similar to those at the young plant stage. In mature green lettuce, the orange spectral treatment resulted in a 15% higher shoot dry weight and a 12% greater total leaf area compared to the red spectral treatment (**Figure 4A**). Shoot dry weights in the O+FR and R+FR treatments were similar, but both were significantly higher (by 15 to 35%) than those in the orange and red spectral treatments (**Figure 4A**). The O+FR treatment resulted in the highest total leaf area in green lettuce, followed by R+FR, orange, and red spectral treatments (**Figure 4B**). LMA was lowest under the O+FR treatment (**Figure 4C**), which had the lowest PSS.

In mature red lettuce plants, the orange spectral treatment led to a significantly higher total leaf area (by 10%) compared to the red spectral treatments, although no significant differences were found in shoot dry weight or LMA (**Figures 4D–F**). Consistent with the responses observed at the young plant stage, partial substitution with FR photons had a more pronounced effect on plant growth and morphology than replacing red photons entirely with orange photons (**Figures 4D–F**).

### 3.2 Projected leaf area

**Figure 5:**
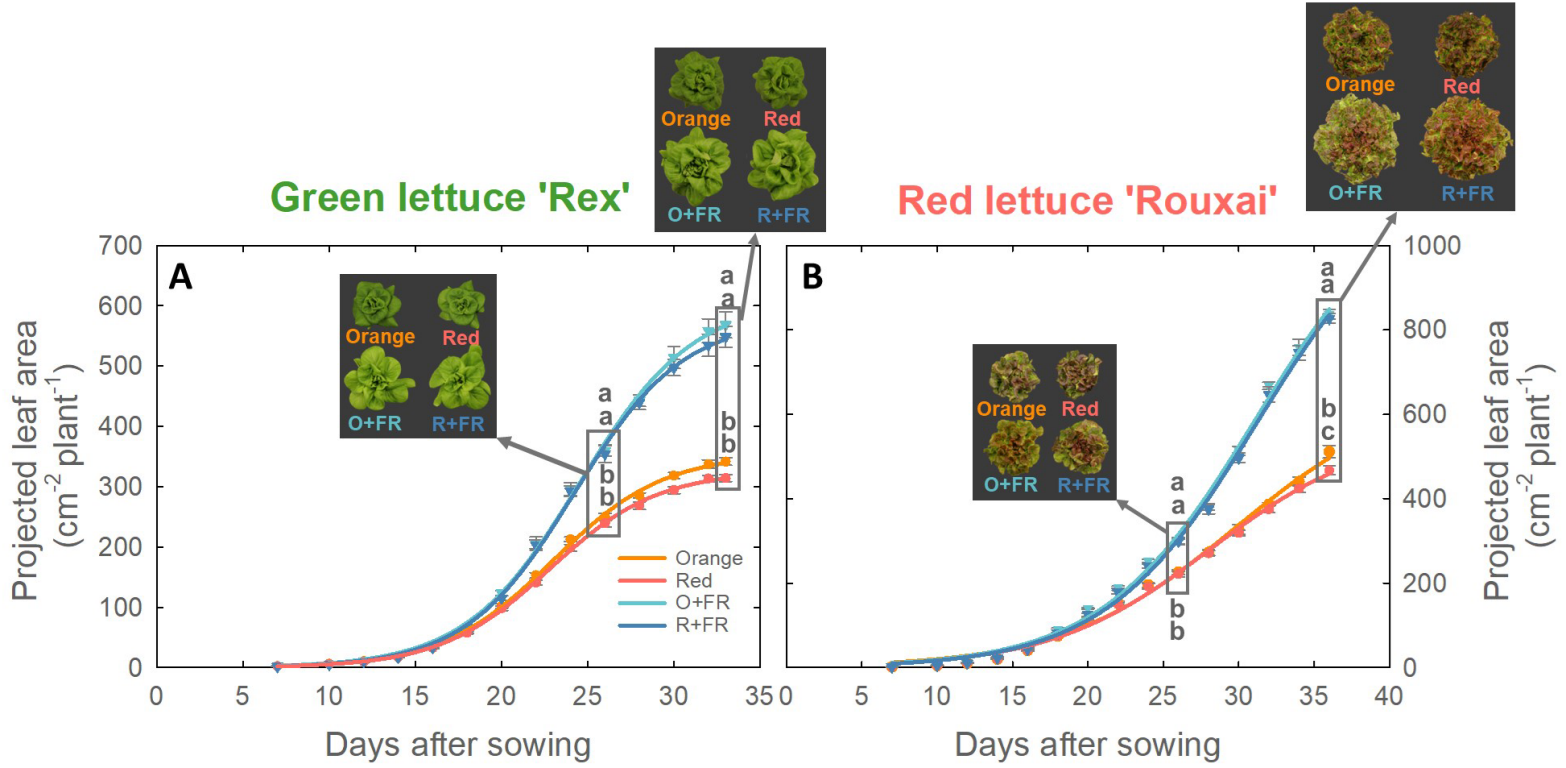
Projected leaf area of green lettuce ‘Rex’ **(A)** and red lettuce ‘Rouxai’ **(B)** grown under four light spectral treatments: Orange (B_50_G_25_O_175_), Red (B_50_G_25_R_175_), O+FR (B_50_G_25_O_137.5_FR_37.5_), and R+FR (B_50_G_25_R_137.5_FR_37.5_). B, G, O, R, and FR stand for blue, green, orange, red, and far-red light, respectively. The subscript numbers indicate the photon flux density of each waveband in µmol m^-2^ s^-1^. Data represent mean ± SE (*n* = 8; 4 plants per treatment × 2 replications). Different letters denote significant treatment differences according to Duncan’s multiple range test at *P <* 0.05.

Projected leaf area (PLA) of both green and red lettuce plants grown under all four spectral treatments exhibited a sigmoidal growth pattern (**Figure 5**). In green lettuce, no significant differences in PLA were observed between treatments with orange or red photons, regardless of FR substitution (**Figure 5A**). However, FR-substituted treatments (O+FR and R+FR) resulted in significantly larger PLA than the non-FR substituted treatments (orange and red) at both harvest times (26 and 33 DAS). In red lettuce, the orange spectral treatment resulted in a 9.5% greater PLA than the red spectral treatment at the final harvest, and FR-substituted treatments exhibited significantly greater PLA compared to the non-FR substituted treatments at both 26 and 36 DAS (**Figure 5B**).

### 3.3 Pigmentation

**Figure 6:**
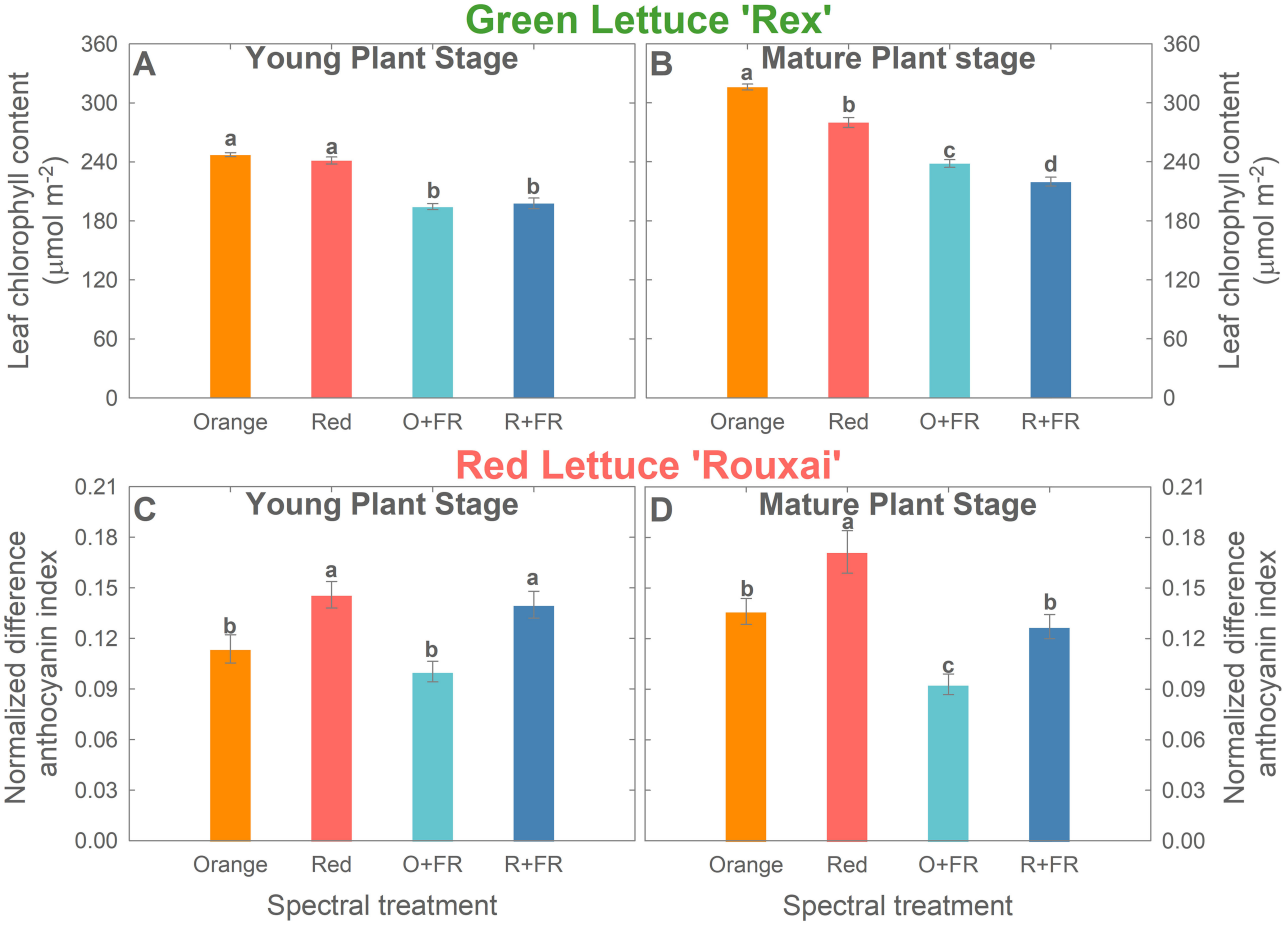
Leaf chlorophyll content of green lettuce ‘Rex’ **(A, B)** and normalized difference anthocyanin index of red lettuce ‘Rouxai’ **(C, D)** at young plant stage [26 days after sowing (DAS)] and mature plant stage (33 DAS for green lettuce and 36 DAS for red lettuce). Plants were grown under four light spectral treatments: Orange (B_50_G_25_O_175_), Red (B_50_G_25_R_175_), O+FR (B_50_G_25_O_137.5_FR_37.5_), and R+FR (B_50_G_25_R_137.5_FR_37.5_). B, G, O, R, and FR stand for blue, green, orange, red, and far-red light, respectively. The subscript numbers indicate the photon flux density of each waveband in µmol m^-2^ s^-1^. Data represent mean ± SE (*n* = 8; 4 plants per treatment per harvest × 2 replications). Different letters denote significant treatment differences according to Duncan’s multiple range test at *P <* 0.05.

*Chlorophyll content.* At the young plant stage (26 DAS), leaf chlorophyll content in green lettuce ‘Rex’ showed no significant differences between treatments with orange or red photons, regardless of the presence of FR (**Figure 6A**). However, FR-substituted treatments showed significantly lower chlorophyll content compared to the non-FR-substituted treatments.

At the mature plant stage, replacing red photons with orange photons increased chlorophyll content in green lettuce, both with and without FR substitution (**Figure 6B**). Among the four spectral treatments, the orange spectral treatment resulted in the highest chlorophyll content, followed by the red spectral treatment (11.4% reduction), the O+FR treatment (24.6% reduction), and the R+FR treatment (30.5% reduction) (**Figure 6B**).

*Anthocyanins*. Orange photons were less effective than red photons in promoting anthocyanin accumulation in red lettuce ‘Rouxai’, regardless of the presence of FR photons, as indicated by the image-based analysis of normalized difference anthocyanin index (NDAI) (**Figures 6C, D**; **Supplementary Figure 1**). At the young plant stage, red lettuce plants grown under the orange treatment had a 25.9% lower NDAI than those under the red treatment, while plants grown under the O+FR treatments showed a 36.9% lower NDAI than those under the R+FR treatment (**Figure 6C**). However, FR substitution did not result in significant differences in NDAI, when comparing the orange and O+FR treatments or the red and R+FR treatments (**Figure 6C**). At the mature plant stage, both the replacement of red photons with orange photons and the partial substitution with FR photons led to a decrease in NDAI (**Figure 6D**). The red spectral treatment resulted in the highest anthocyanin index, whereas the orange, R+FR, and O+FR treatments showed reductions of 20.6%, 25.8%, and 45.8%, respectively (**Figure 6D**).

### 3.4 Leaf photon absorption, transmittance, and reflectance

**Figure 7:**
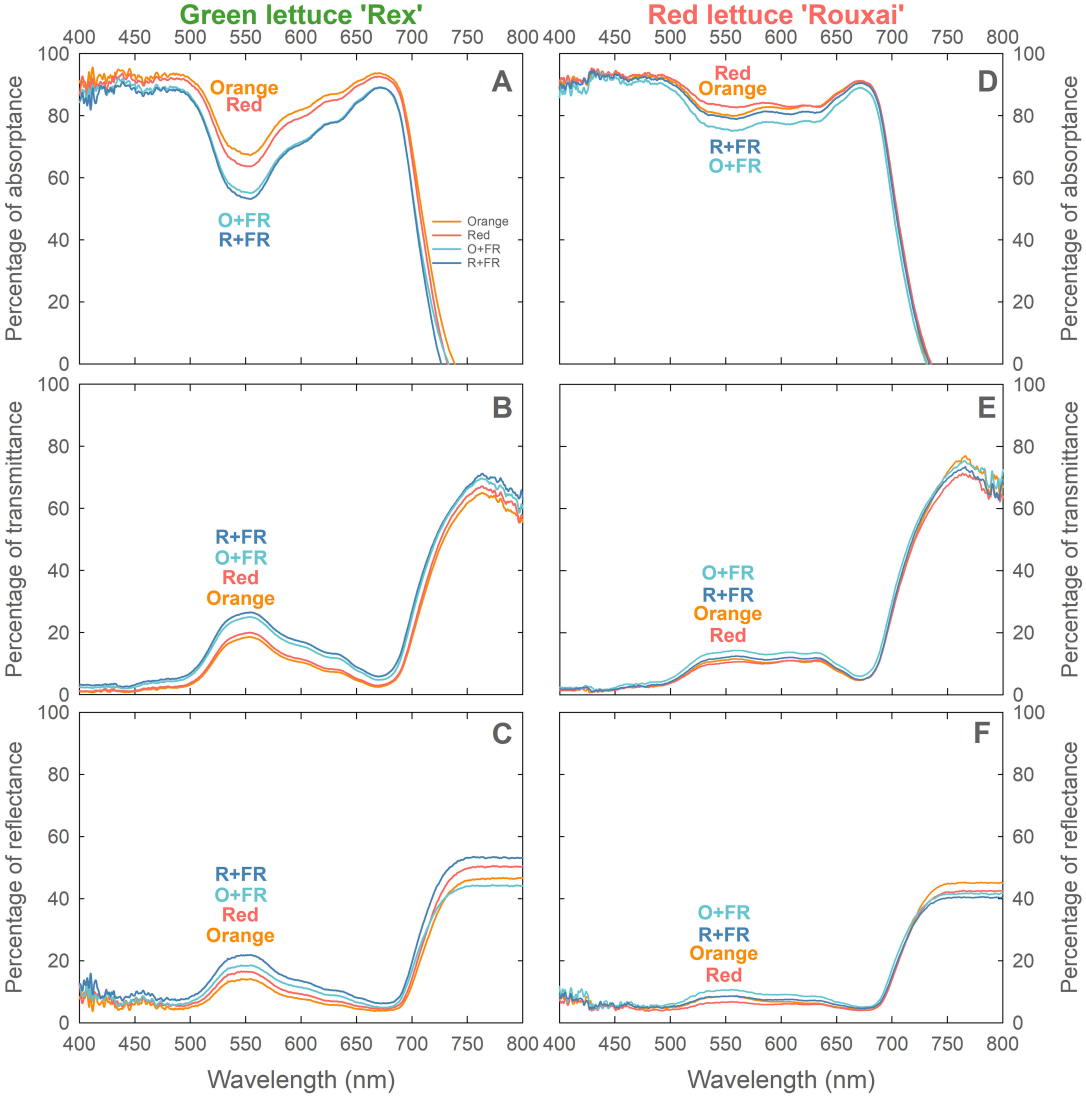
Leaf absorptance **(A, D)**, transmittance **(B, E)**, and reflectance **(C, F)** of green lettuce ‘Rex’ **(A, B)** and red lettuce ‘Rouxai’ **(C, D)** grown under four spectral treatments: Orange (B_50_G_25_O_175_), Red (B_50_G_25_R_175_), O+FR (B_50_G_25_O_137.5_FR_37.5_), and R+FR (B_50_G_25_R_137.5_FR_37.5_). B, G, O, R, and FR stand for blue, green, orange, red, and far-red light, respectively. The subscript numbers indicate the photon flux density of each waveband in µmol m^-2^ s^-1^. Measurements were made using the uppermost fully expanded leaves at the end of the experiment (*n* = 6; 3 plants per treatment × 2 replications).

Green lettuce ‘Rex’ grown under the orange spectral treatment exhibited the highest leaf light absorptance (and the lowest transmittance and reflectance) across the photosynthetically active radiation waveband (400–700 nm), followed by plants grown under the red, O+FR, and R+FR treatments (**Figures 7A–C**). Plants grown in the FR-substituted treatments showed reduced light absorptance and increased transmittance and reflectance compared to those under the non-FR-substituted treatments. By contrast, red lettuce ‘Rouxai’ (**Figures 7D–F**) grown under the red spectral treatment had the highest leaf light absorptance (and the lowest transmittance and reflectance), followed by plants grown under the orange, R+FR, and O+FR treatments. Red lettuce showed higher light absorption in the green region (500–600 nm) than green lettuce. Similar to responses observed in green lettuce, FR-substitution resulted in reduced leaf light absorptance and increased transmittance and reflectance in red lettuce.

### 3.5 Photosynthetic responses to orange and red photons

**Figure 8:**
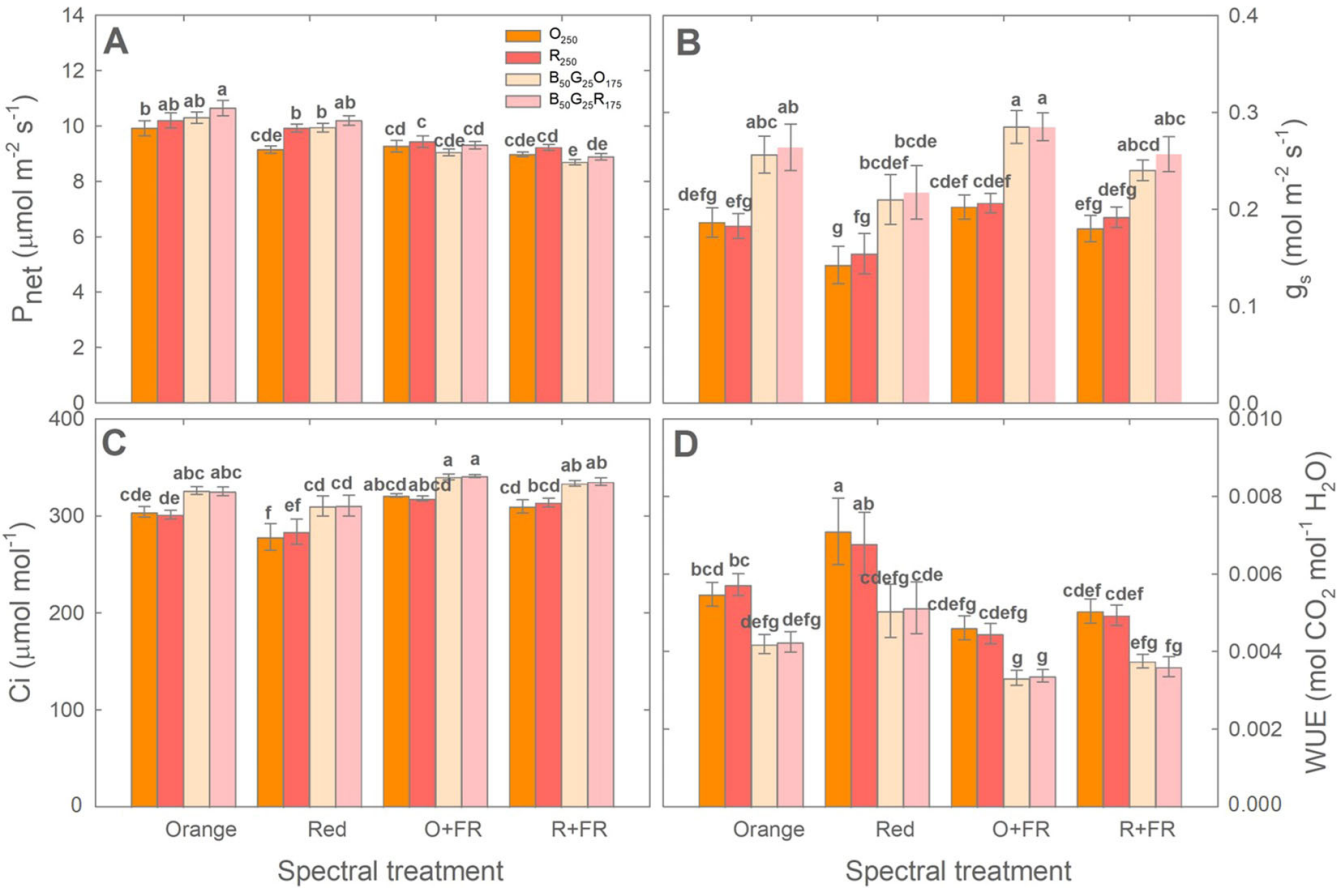
Net photosynthetic rate (Pnet; **A)**, stomatal conductance (gs; **B)**, intercellular CO_₂_ concentration (Ci; **C)**, and water use efficiency (WUE; **D)** of green lettuce ‘Rex’ measured under four light conditions [monochromatic orange light (O_250_), monochromatic red light (R_250_), combined blue, green, and orange light (B_50_G_25_O_175_), and combined blue, green, and red light (B_50_G_25_R_175_)]. Plants were grown under four light spectral treatments: Orange (B_50_G_25_O_175_), Red (B_50_G_25_R_175_), O+FR (B_50_G_25_O_137.5_FR_37.5_), and R+FR (B_50_G_25_R_137.5_FR_37.5_). B, G, O, R, and FR stand for blue, green, orange, red, and far-red light, respectively. The subscript numbers indicate the photon flux densities in µmol m^-2^ s^-1^. Data represent mean ± SE (*n* = 6; 3 plants per treatment × 2 replications). Different letters denote significant treatment differences according to Duncan’s multiple range test at *P <* 0.05.

Both acclimation to growth light spectral treatments and the light conditions during photosynthesis measurements influenced the photosynthetic activity of green lettuce ‘Rex’ (**Figure 8**). Across all four measurement light conditions (O_250_, R_250_, B_50_G_25_O_175_, and B_50_G_25_R_175_), plants grown without FR light substitution (i.e., orange and red spectral treatments) generally showed higher P_net_ than those grown under FR-substituted treatments (O+FR and R+FR) (**Figure 8A**). Within each growth spectral treatment, photosynthesis measurements under monochromatic orange light (O_250_) resulted in similar P_net_, g_s_, C_i_ and WUE compared to measurements under monochromatic red light (R_250_), except that P_net_ under R_250_ was significantly higher (by 8.5%) than that under O_250_ in plants grown under the red spectral treatment.

Across all four growth spectral treatments, the inclusion of blue and green in the photosynthesis measurement light spectrum (B_50_G_25_O_175_ and B_50_G_25_R_175_) resulted in higher g_s_ compared to monochromatic O_250_ or R_250_. This increased gs tended to correspond to higher P_net_ (in plants grown without FR light only), higher C_i_, and lower WUE (**Figure 8**). When measured under monochromatic orange light (O_250_), plants grown under the orange treatment showed higher P_net_ (by 8%) and C_i_ (by 9%), similar g_s_, but lower WUE (by 23%) compared to those grown under the red treatment. However, under other measurement light conditions (R_250_, B_50_G_25_O_175_ and B_50_G_25_R_175_), P_net_, g_s_, C_i_, and WUE did not differ significantly between plants grown under orange and red spectral treatments or between the O+ FR and R+FR treatments (**Figure 8**).

### 3.6 Quantum yield of orange and red photons

The estimated quantum yield under the monochromatic O_250_ measurement light was significantly higher than under monochromatic R_250_ measurement light in plants grown under the orange, O+FR, and R+FR treatments, by 13%, 10%, and 9%, respectively (**Table 1**). However, no significant difference was observed in plants grown under the red spectral treatment (**Table 1**).

**Table 1:** Estimated quantum yield for CO_2_ assimilation under monochromatic orange (623 nm) and red (660 nm) measurement light.

Light spectral treatment^1^	Photosynthetic measurement light^2^	Gross photosynthesis^3^ (µmol m^-2^ s^-1^)	Quantum Yield^4^ (µmol mol^-1^)
B_50_G_25_O_175_ (Orange)	Orange (623 nm)	10.82 ± 0.28 ab	0.0532 ± 0.0011 a
	Red (660 nm)	11.10 ± 0.29 a	0.0471 ± 0.0012 b
B_50_G_25_R_175_ (Red)	Orange	10.00 ± 0.16 c	0.0507 ± 0.0005 b
	Red	10.80 ± 0.16 ab	0.0484 ± 0.0006 b
B_50_G_25_O_137.5_FR_37.5_ (O+FR)	Orange	10.12 ± 0.22 c	0.0535 ± 0.0010 a
	Red	10.30 ± 0.23 bc	0.0486 ± 0.0010 b
B_50_G_25_R_137.5_FR_37.5_ (R+FR)	Orange	9.84 ± 0.10 c	0.0522 ± 0.0009 a
	Red	10.09 ± 0.13 c	0.0478 ± 0.0006 b

Measurements were made 2–6 days before the final harvest (27–31 days after sowing).

^1^Light-emitting diodes (LEDs) were used to provide blue (B), green (G), orange (O), red (R), and far-red (FR) photons. The subscript after each waveband indicates its photon flux density in μmol m^-2^ s^-1^.

^2^The intensity of monochromatic orange or red light was 250 µmol m^-2^ s^-1^.

^3,4^Data represent the mean ± S.E (n = 6). Different letters indicate significant differences at *P* < 0.05.

## 4 Discussion

### 4.1 Orange versus red photons: differences in light penetration and cryptochrome-mediated morphological responses

This study investigated the morphological and physiological responses of two lettuce cultivars to orange (623 nm) versus red (660 nm) photons, with or without the presence of far-red photons. In the absence of far-red photons, green lettuce ‘Rex’ exhibited significantly greater shoot biomass and leaf expansion under the orange spectral treatment at both the young and mature plant stage, whereas red lettuce ‘Rouxai’ showed significantly enhanced leaf expansion only at maturity, without a corresponding increase in shoot biomass. The PSS values, which indicate phytochrome activity, were the same (0.88) in both treatments. These morphological differences in response to orange versus red photons may be attributed to (1) reduced cryptochrome activity under orange photons and (2) improved light penetration of orange photons.

Cryptochrome photoreceptors perceive both blue and green/yellow/orange photons, which have opposing effects on the cryptochrome activation state. Blue photons activate cryptochromes by converting the inactive oxidized flavin (FAD_ox_) form to the active semiquinone (FADH˚) form (Bouly et al., 2007; Bugbee, 2016; Battle et al., 2020; Zhen et al., 2022). This activation results in cryptochrome-mediated morphological changes, including reduced stem elongation and leaf expansion, ultimately leading to decreased biomass accumulation in various species such as lettuce, basil, cucumber, soybean, pepper, and tomato (Snowden et al., 2016; Meng et al., 2019, 2020; Kusuma et al., 2021; Kang et al., 2022; Zhen et al., 2022; Kang et al., 2024; Zhu et al., 2024). Green/yellow/orange photons (530–630 nm) revert the active FADH˚ form to its fully reduced inactive state (FADH^-^) (Bouly et al., 2007; Battle et al., 2020). This reversion downregulates cryptochrome activity and promotes expansion and elongation growth, which are often associated with biomass increases (Snowden et al., 2016; Meng et al., 2020; Kusuma et al., 2021; Kang et al., 2024). In our experiment, the fractions of blue and green photons were fixed at 20% and 10%, respectively, across all treatments; only the proportion of orange (623 nm) and red photons (660 nm) were varied. Although the orange and red photons emitted by the LEDs used in this study fall outside the absorption peaks of cryptochromes, Bouly et al. (2007) reported FADH˚ absorption in the wavelength region above 600 nm, with absorption gradually decreasing and becoming minimal beyond 650 nm. These findings suggest that the increased leaf expansion observed under orange photons (**Figures 3B, E**, **Figures 4B, E**) may be due to cryptochrome deactivation.

Another contributing factor may be the greater light penetration of orange photons. Massa et al. (2015) reported that orange photons (631 nm) had higher leaf transmittance (by around 6%) than red photons (660 nm) in lettuce, at both low (225 µmol m^-2^ s^-1^) and high (420 µmol m^-2^ s^-1^) light intensities. Leaves in the upper canopy absorb more than 90% of red and blue photons, whereas orange and green photons can penetrate deeper and sustain photosynthesis in lower leaf cell layers and in shaded leaves within the canopy (Terashima et al., 2009; Brodersen and Vogelmann, 2010; Massa et al., 2015; Dieleman et al., 2019; Liu and van Iersel, 2021). Dieleman et al. (2019) further showed that supplementing sunlight with orange light (627 nm) resulted in the highest shoot dry weight in greenhouse-grown tomatoes, compared to red light supplementation (660 nm) at the same light intensity (225 µmol m^-2^ s^-1^), likely due to improved light distribution within the canopy. However, Wollaeger and Runkle (2013) observed no differences in seedling growth of ornamental and annual crops, including impatiens, marigold, petunia, and tomato, when grown under orange (634 nm) versus red (660 nm) photons, both provided with a background of 10% blue and 10% green light. The effects of orange photons may be crop or growth stage-specific and more pronounced in larger canopies with overlapping leaves.

### 4.2 Interactions of orange and red photons with far-red light

The inclusion of far-red photons (15% of TPFD) in the O+FR and R+FR treatments significantly increased shoot biomass and leaf expansion in both cultivars, consistent with previous reports of far-red–induced morphological responses (Kim et al., 2004; Pierik and de Wit, 2013; Meng et al., 2019; Zhen and Bugbee, 2020a). However, no significant growth differences were observed between the O+FR and R+FR treatments, despite the lower PSS value in O+FR (0.78 vs. 0.82 in R+FR). This may reflect complex cross-regulation between cryptochromes and phytochromes. Far-red light is an environmental cue for shade and is sensed via phytochromes (Franklin and Whitelam, 2007). Orange photons may elicit shade-like responses by de-activating cryptochromes, in a manner similar to green photons, though less efficiently (Bouly et al., 2007). Previous studies have shown that shade responses induced by far-red photons can be enhanced by green photons, suggesting a complementary role of green and far-red photons in triggering shade responses (Wang et al., 2015). However, no such synergism was observed between orange and far-red photons in O+FR treatments compared to R+FR treatment. The similar growth responses under the O+FR and R+FR treatments suggest that strong phytochrome inactivation by far-red may override any differences in cryptochrome-mediated morphological differences induced by orange photons.

### 4.3 Effects of orange photons on pigmentation

Orange photons influenced leaf pigmentation. Green lettuce grown under the orange spectral treatment exhibited higher chlorophyll content and leaf light absorptance at the mature plant stage compared to the red spectral treatment, while LMA values were similar, indicating comparable leaf thickness (**Figures 4C**, **6B** and **7A**). Although studies examining the effects of orange photons remain limited, our findings contrast with previous research on green light, which has shown that cryptochrome deactivation by green photons is typically associated with reduced pigment accumulation and thinner leaves (Bouly et al., 2007; Snowden et al., 2016; Cammarisano et al., 2021; Kusuma et al., 2021; Park and Runkle, 2023; Kang et al., 2024). Orange may similarly de-activate cryptochromes, yet our results showed the opposite trend. Nonetheless, our results align with previous studies on the effects of orange photons. Dieleman et al. (2019) found that tomato plants supplemented with orange light under a background of sunlight developed thicker leaves, while maintaining similar chlorophyll levels compared to those grown under supplemental red light. Similarly, Wollaeger and Runkle (2013) observed trends toward increased chlorophyll content under orange photons (peak at 634 nm) compared to red photons (660 nm) in several ornamental and vegetable seedlings, although the differences were not statistically significant.

Treatments with far-red substitution (O+FR and R+FR) resulted in significantly reduced leaf light absorptance compared to non-FR substituted treatments due to the lower chlorophyll contents and thinner leaves (**Figures 3**, **4**, **6**) induced by far-red light (Gommers et al., 2013; Zhen and Bugbee, 2020a).

Red lettuce grown under orange photons (in both the orange and O+FR treatments) showed significantly lower anthocyanin accumulation, as indicated by lower NDAI values, compared to plants grown under red photons (both the red and R+FR treatments) (**Figures 5B**, **6D**, and **7D**). This is consistent with previous findings that cryptochrome activation increases anthocyanin accumulation, whereas its deactivation leads to reduced anthocyanin levels (Bouly et al., 2007; Snowden et al., 2016; Kusuma et al., 2021; Kang et al., 2022; Park and Runkle, 2023; Zhu et al., 2024). Furthermore, the O+FR treatment resulted in the lowest NDAI among the four spectral treatments, which due to its PSS value being the lowest among the four treatments.

### 4.4 Cultivar specific responses to orange photons

Our results indicate cultivar-specific responses to orange versus red photons. Green lettuce ‘Rex’ showed increased leaf expansion, chlorophyll content, and biomass accumulation under orange photons, particularly at the mature plant stage. In contrast, the most noticeable response in red lettuce ‘Rouxai’ under the orange spectral treatment was a reduction in anthocyanin accumulation with no significant difference in biomass. However, these cultivar-specific responses were diminished in both cultivars in the presence of far-red photons, as plants exhibited similar growth, morphology, and pigmentation under both O+FR and R+FR treatments.

### 4.5 Photosynthetic activities of orange versus red photons

Studies by McCree (1971) and Inada (1976) showed that orange photons (peak ~625 nm) have the highest relative quantum yield, i.e., moles of CO_2_ fixed per mole of absorbed photons, within the PAR region followed by red photons (peak at around 675 nm), which exhibit about 10% lower quantum yield. These measurements were conducted under low light intensity conditions (below 150 µmol m^-2^ s^-1^). In our study, when evaluated on an incident photon basis, P_net_ of green lettuce ‘Rex’ measured under orange photons (O_250_ and B_50_G_25_O_175_) were generally lower than under red photons (i.e., O_250_ versus R_250_ or B_50_G_25_O_175_ versus B_50_G_25_R_175_), regardless of the growth light spectral treatment (**Figure 8**). However, estimated quantum yields were approximately 10% higher under the orange measurement light (O_250_) than the red measurement light (R_250_) when accounting for the differences in leaf light absorption (**Table 1**). Our quantum yield data were consistent with previous findings by McCree (1971) and Inada (1976).

It is worth noting that acclimation to the growth light spectral treatments affected the efficiency at which plants used orange versus red photons for photosynthesis. Specifically, plants grown under the orange spectral treatment used orange light more efficiently than those grown under the red spectral treatment, as indicated by the higher P_net_ under O_250_ in plants acclimated to orange photons (**Figure 8A**).

When measured under mixed light conditions containing blue and green photons (B_50_G_25_R_175_ and B_50_G_25_O_175_), plants consistently showed increased stomatal conductance compared to measurements under orange or red light (**Figure 8B**). However, those increases in stomatal conductance corresponded to higher P_net_ only in plants grown without FR light (**Figure 8A**). Blue photons are well known to induce stomatal opening, which increases internal CO_2_ concentration and thereby enhancing photosynthetic rate. Nonetheless, blue-light-induced stomatal opening does not always result in higher photosynthetic rate. For example, Zhen and Bugbee (2020b) reported that although higher blue light fractions increased stomatal conductance in sunflower, leaf photosynthetic rate generally decreased at high blue light fractions, leading to reduced water use efficiency.

Consistent with previous findings, the inclusion of FR light in the growth light spectrum generally resulted in lower leaf P_net_ compared to plants grown without FR light (**Figure 8A**). This reduction is likely at least in part due to decreased chlorophyll content and reduced leaf light absorptance as a result of acclimation to FR-enriched conditions (Kalaitzoglou et al., 2019; Zhen et al., 2019).

### 4.6 Fixture efficacy of orange versus red LEDs

Practical applications in controlled environment crop lighting systems must also account for the efficacy of LED fixtures in converting electricity into photosynthetic light. Current red LEDs (peak ~660–670 nm) are typically more energy-efficient than orange LEDs (peak ~620–630 nm) (Wollaeger and Runkle, 2013; Kusuma et al., 2022). As a result, although orange light may promote leaf expansion and support a higher photosynthetic quantum yield, its lower fixture efficacy can lead to increased energy costs for crop lighting. Therefore, optimizing light spectral quality for crop production may involve a tradeoff between maximizing plant growth, physiological parameters, and quality attributes—such as photosynthetic efficiency and desirable morphology—and minimizing electrical energy use of the light fixtures. Cost-effective lighting strategies must consider both plant growth responses and electric consumption.

## 5 Conclusions

Our results showed that the orange spectral treatment (peak at 623 nm) resulted in similar or greater shoot biomass and total leaf area in both green lettuce ‘Rex’ and red lettuce ‘Rouxai’ compared to the red spectral treatment (peak at 660 nm), likely due to partial cryptochrome deactivation by orange photons. However, the anthocyanin content in ‘Rouxai’ was lower under the orange spectral treatment. The estimated leaf quantum yield under monochromatic orange measurement light was generally higher than that under red measurement light. In both cultivars, the inclusion of far-red photons (O+FR and R+FR) significantly enhanced plant growth, but no significant growth differences were observed between the O+FR and R+FR treatments. Although orange light may promote plant growth, its lower fixture efficacy compared to red LEDs could increase energy costs—an important consideration for controlled environment applications.

## Data Availability

The original contributions presented in the study are included in the article/**Supplementary Material**. Further inquiries can be directed to the corresponding author.
