# Supplementary material for: Orange photons (623 nm) resulted in similar or greater lettuce growth than red photons (660 nm): comparative effects on morphology, photon capture, and photosynthesis
Source: Front Plant Sci. 2025 Jul 30;16:1653524. doi: 10.3389/fpls.2025.1653524 (PMC12343277; doi:10.3389/fpls.2025.1653524)
Supplement: Supplementary file 1 [file DataSheet1.pdf]

Supplementary Material

Orange Photons (623 nm) Resulted in Similar or Greater Lettuce Growth than Red Photons (660 nm): Comparative Effects on Morphology, Photon capture, and Photosynthesis

Seonghwan Kang and Shuyang Zhen*
Department of Horticultural Sciences, Texas A&M University, College Station, TX, United States

1 Supplementary Figures

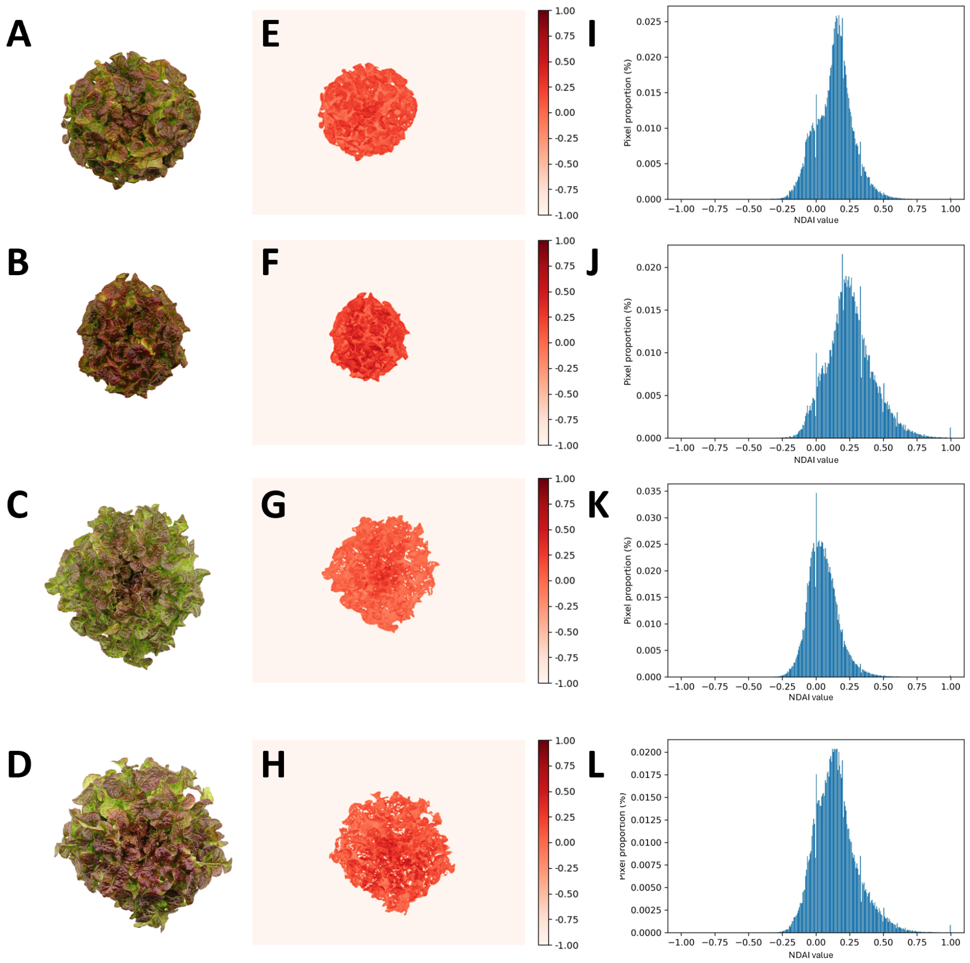

Supplementary Figure 1. Image analysis of normalized difference anthocyanin index (NDAI) following the method described by Kim and van Iersel (2023). Panels A-D show representative photos of red lettuce 'Rouxai' grown under four light spectral treatments: (A) Orange ($B_{50}G_{25}O_{175}$), (B) Red ($B_{50}G_{25}R_{175}$), (C) O+FR ($B_{50}G_{25}O_{137.5}FR_{37.5}$), and (D) R+FR ($B_{50}G_{25}R_{137.5}FR_{37.5}$). The corresponding NDAI images (E-H) and histograms (I-L) are included for each treatment. B, G, O, R, and FR stand for blue, green, orange, red, and far-red light, respectively. The subscript numbers indicate the photon flux density of each waveband in μmol m^{-2} s^{-1}.

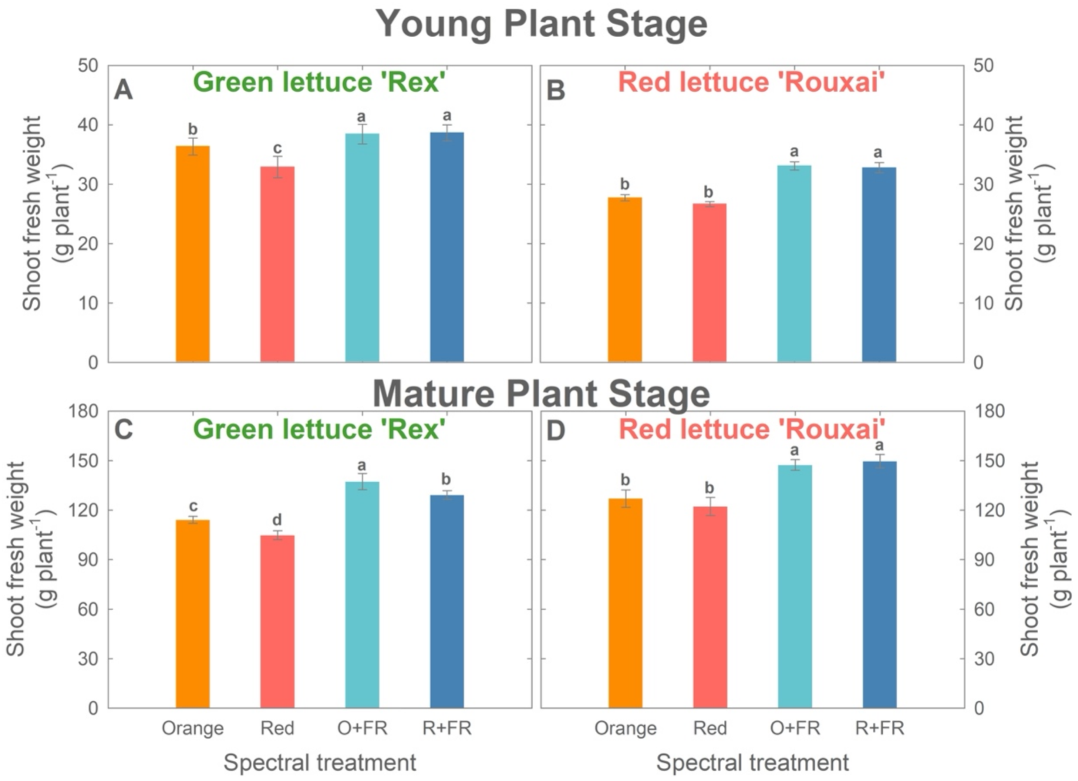

Supplementary Figure. 2. Shoot fresh weight of green lettuce 'Rex' (A, C) and red lettuce 'Rouxai' (B, D) at the young plant stage (26 days after sowing; A-B) and the mature plant stage (33 days after sowing for green lettuce and 36 days after sowing for red lettuce; C-D). Plants were grown under four light spectral treatments: Orange ($B_{50}G_{25}O_{175}$), Red ($B_{50}G_{25}R_{175}$), O+FR ($B_{50}G_{25}O_{137.5}FR_{37.5}$), and R+FR ($B_{50}G_{25}R_{137.5}FR_{37.5}$). B, G, O, R, and FR stand for blue, green, orange, red, and far-red light, respectively. The subscript numbers indicate the photon flux density of each waveband in µmol m^{-2} s^{-1}. Data represent mean ± SE (n = 8; 4 plants per treatment per harvest × 2 replications). Different letters denote significant treatment differences according to Duncan's multiple range test at $P < 0.05$.

2 Supplementary Tables

Supplementary Table 1. Light intensity and spectral characteristics of the four spectral treatments. Light-emitting diodes (LEDs) were used to provide blue (B), green (G), orange (O), red (R), and far-red (FR) photons. The subscript after each waveband indicates its photon flux density in μmol m^{-2} s^{-1}.

Light spectral treatment	Photon flux density (μmol m^{-2} s^{-1}) provided by each type of LEDs					
	Blue LEDs (peak at 444 nm)	Green LEDs (peak at 536 nm)	Orange LEDs (peak at 623 nm)	Red LEDs (peak at 660 nm)	Far-red LEDs (peak at 730 nm)	TPFD^x (μmol m^{-2} s^{-1})
$B_{50}G_{25}O_{175}$ (Orange)	50.9^y ± 1.8^z	24.9 ± 1.8	174.6 ± 3.9	N/A	N/A	250.9 ± 5.2
$B_{50}G_{25}R_{175}$ (Red)	51.3 ± 2.8	24.3 ± 2.5	N/A	175.0 ± 6.1	N/A	250.6 ± 9.2
$B_{50}G_{25}O_{137.5}FR_{37.5}$ (O+FR)	50.6 ± 3.9	24.7 ± 1.4	136.5 ± 2.8	N/A	36.1 ± 1.5	250.8 ± 5.7
$B_{50}G_{25}R_{137.5}FR_{37.5}$ (R+FR)	50.1 ± 1.7	24.4 ± 1.0	N/A	138.1 ± 3.4	37.4 ± 1.3	251.2 ± 5.5

TPFD^x: total photon flux density integrated from 400 to 800 nm.

mean^y: values represent the average of two replications, each consisting of 16 light measurements taken under the treatment area using a spectroradiometer.

SD^z: standard deviation.
